# Arctic tidal current atlas

**DOI:** 10.1038/s41597-020-00578-z

**Published:** 2020-08-21

**Authors:** Till M. Baumann, Igor V. Polyakov, Laurie Padman, Seth Danielson, Ilker Fer, Markus Janout, William Williams, Andrey V. Pnyushkov

**Affiliations:** 1 International Arctic Research Center and College of Natural Science and Mathematics, University of Alaska Fairbanks (UAF), Fairbanks, AK USA; 2 Finnish Meteorological Institute, Helsinki, Finland; 3 Earth and Space Research, Corvallis, OR USA; 4 College of Fisheries and Ocean Sciences, UAF, Fairbanks, AK USA; 5 Present Address: Geophysical Institute, University of Bergen and Bjerknes Centre for Climate Research, Bergen, Norway; 6 Alfred Wegener Institute, Bremerhaven, Germany; 7 Fisheries and Oceans Canada, Sydney, BC Canada; 8 International Arctic Research Center, UAF, Fairbanks, AK USA; 9 Global Institution for Collaborative Research and Education, Hokkaido University, Hokkaido, Japan

**Keywords:** Planetary science, Physical oceanography

## Abstract

Tidal and wind-driven near-inertial currents play a vital role in the changing Arctic climate and the marine ecosystems. We compiled 429 available moored current observations taken over the last two decades throughout the Arctic to assemble a pan-Arctic atlas of tidal band currents. The atlas contains different tidal current products designed for the analysis of tidal parameters from monthly to inter-annual time scales. On shorter time scales, wind-driven inertial currents cannot be analytically separated from semidiurnal tidal constituents. Thus, we include 10–30 h band-pass filtered currents, which include all semidiurnal and diurnal tidal constituents as well as wind-driven inertial currents for the analysis of high-frequency variability of ocean dynamics. This allows for a wide range of possible uses, including local case studies of baroclinic tidal currents, assessment of long-term trends in tidal band kinetic energy and Arctic-wide validation of ocean circulation models. This atlas may also be a valuable tool for resource management and industrial applications such as fisheries, navigation and offshore construction.

## Abstract (summary)

**Table:** 

Measurement(s)	ocean current
Technology Type(s)	Gauge or Meter Device • digital curation
Factor Type(s)	time • current • geographic location • tidal current
Sample Characteristic - Environment	ocean
Sample Characteristic - Location	Arctic Ocean region

Machine-accessible metadata file describing the reported data: 10.6084/m9.figshare.12581435

## Background & Summary

Tidal currents are often the dominant source of current variability and play an important role in shaping the Arctic Ocean hydrography and sea ice cover^1–3^. Tidal currents are also a key element shaping the marine ecosystem with impacts ranging from creating the habitat of the intertidal zone to mixing of nutrients. Furthermore, information about tidal currents is used for many practical applications, such as navigation, fisheries and marine structures and operations.

Barotropic tidal models^1,4^ based on the depth-integrated momentum and continuity equations provide ocean surface height and depth-averaged currents for major tidal constituents throughout the Arctic. These comparatively simple models show very little tidal activity (<0.5 cm/s) in the central Arctic deep basins, but strong amplitudes (>10 cm/s) over portions of the continental shelves and slopes.

Where barotropic tidal currents flow across steep slopes or rough topography in the presence of stratification, energy can be converted from barotropic to baroclinic (internal) tides whose energy finally dissipates in mixing processes^5^. The importance of baroclinic tidal processes was highlighted, for example, by Luneva *et al*.^3^, who found that the addition of tidal currents to an atmospherically forced three-dimensional simulation reduced pan-Arctic sea ice volume by ~15%. The authors attributed this sea ice reduction to the entrainment of warm subsurface Atlantic Water into the cold near-surface waters by mixing caused by increased surface stresses and by upper-ocean shear instabilities from the combination of baroclinic tides and the atmospherically forced three-dimensional circulation. In contrast to barotropic tides, the generation, propagation and dissipation of baroclinic tidal waves are sensitive to stratification, mean flow, and energy losses through friction and mixing within the water column. They may, therefore, change substantially with variations in the background ocean state associated with weather-band and seasonal changes in forcing, ocean mesoscale variability (e.g., eddies) and as the Arctic Ocean changes on longer time scales e.g.^6^.

Despite the importance of tides for the Arctic Ocean and its sea ice, most ocean general circulation models used for climate projections do not currently include tides. At this time, comprehensive Arctic oceanographic data sets are limited to hydrographic variables (salinity, temperature and density, e.g. The Arctic Ocean Atlas, compiled by the US-Russian Environmental Working Group with data spanning the 1950s to the 1980s^7^) with no direct information about ocean currents. With the increased deployment of moored Acoustic Doppler Current Profilers in the Arctic in the last two decades, high-resolution current observations (predominantly of the upper ocean) have become available. Using these data, detailed analyses of tidal current dynamics have been carried out at several specific locations in the Arctic such as the Beaufort Sea shelf^8^, the Yermak Plateau^9^, the Nares Strait^10^, the Laptev Sea^11^ and the eastern Eurasian Basin^12,13^). These studies emphasize the importance of tidal currents to local ocean dynamics. However, a pan-Arctic perspective on observed 3-D tidal currents is required, both to synthesize and expand our understanding of tidal dynamics and its interactions with hydrography and sea ice, and to validate numerical models. Here we present an atlas of tidal currents from available moored current meter records spanning the past two decades in all sectors of the Arctic Ocean. The aim is to provide a data set enabling regional in-depth analyses of time-depth dependent tidal currents. With information about local baroclinicity, this atlas complements existing altimetry-based products of barotropic tides and provides reference points for evaluating and constraining model simulations. Long time series may be used to identify and analyse trends of tidal-band current dynamics.

## Methods

### Data acquisition and pre-processing

**Fig. 1:**
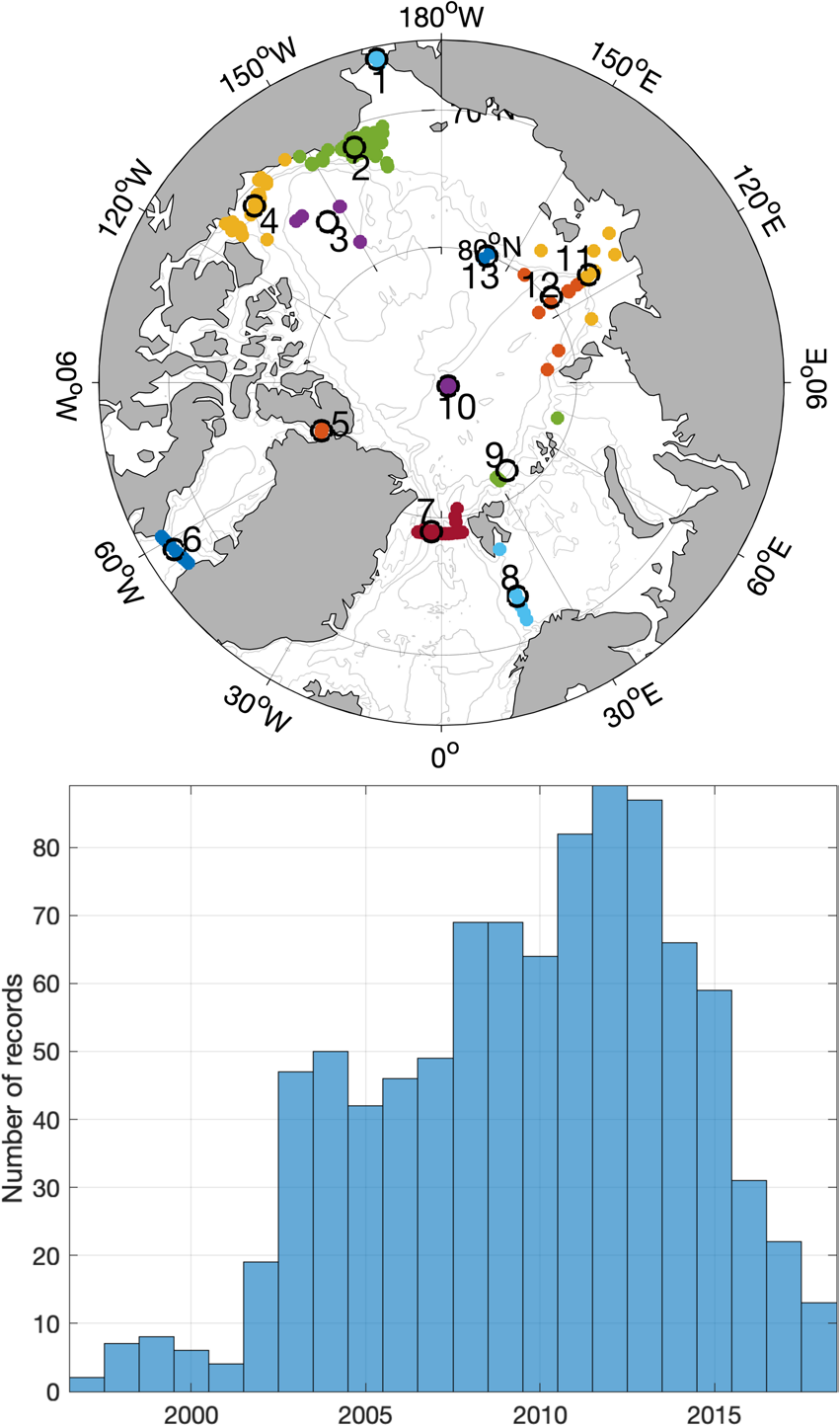
Spatial and temporal distribution of current velocity records contained in the atlas. Top: Map showing locations of the records (coloured dots). Colours indicate grouping utilized for visualizations. Black circles show the centroid location and number of each cluster. Bottom: Histogram of record distribution over time.

One of the targets for this atlas was to collect all available Arctic current profile records of at least 1-year length and 1-h resolution to resolve tidal oscillations. With the help of many colleagues, we gathered 429 records from all sectors of the Arctic (although not homogeneously distributed), spanning the last two decades (Online-only Table 1, Fig. 1).

Most observations were obtained with Acoustic Doppler Current Profilers (ADCPs) from TELEDYNE RD Instruments (TRDI), with the 300 kHz variant being the most commonly used. ADCPs generally provide vertical profiles of horizontal velocity with a vertical resolution and range depending on the instrument’s frequency and set-up. In the Arctic, where there are relatively few backscattering particles outside the shallow shelf regions, typical vertical resolutions range from 0.5 to 5 m with vertical ranges spanning 15 m to ~300 m for 1200 kHz and 75 kHz ADCPs, respectively. Typical temporal resolution of the records is 1 h, although some deployments have resolution of 15–30 minutes. While vertical resolution and range vary substantially between models of different frequencies, the expected accuracies for speeds and directions are generally similar and are given as ±0.5 cm/s and ±2° for vertical averaging bin sizes of 2 m for the 300 kHz ADCPs. Known issues with moored ADCP records are discussed in the section “Technical Validation”. In the Barents Sea Opening region, where ADCP records were sparse, we complemented the atlas with data from Recording Current Meters (RCMs), which work in an analogous fashion to mechanical anemometers and provide point observations of currents at the depth they are moored. Aanderaa RCM7 instruments have a starting velocity of 2 cm/s with expected accuracies of 1 cm/s or 4% of the actual speed (whichever is higher) and the accuracy for the direction is expected to be 5° (Aanderaa Instruments data sheet). While the current speed measured by RCMs is averaged over several observations within a chosen time interval (usually 1 h) around the designated time of measurement, the compass direction is retrieved only once, instantaneously at the designated time.

The data used in this atlas came from many different sources in many different formats. The number of steps required to arrange the data in a common format depended on the original format and state of processing. Generally, the first step was to ensure a uniform grid of time and depth. It was not uncommon for the records to have a drifting clock or otherwise inconsistent time spacing. If the original time vector was not equally spaced throughout the length of the deployment, the data were interpolated onto a synthetic time vector with 1 h time interval. Data gaps of more than 1 h were kept but filled with missing data delimiter “NaN”. If information about time-varying instrument depth was available, either from the pressure sensor of the ADCP itself or Conductivity, Temperature, Depth (CTD) sensors on the same mooring near the ADCP’s depth, the depth of each ADCP bin was adjusted at every time step. The data were then interpolated onto a uniform pressure vector (spreading the whole depth range covered by observations while maintaining the original increment) at each time step. If no pressure record was available, a constant instrument depth at the depth of deployment was used, and all bin depths were assumed to be constant over time. Since some depth information was provided in pressure units (dbar) and others in distance from the surface (meters) without necessarily providing CTD data for conversion, we decided to treat any depth information as pressure. The error associated with this approximation is ~1.1% of the depth of the sensor. Since most records only cover the upper ocean (<100 m depth), the error is small and tends to be less than 1 m. We chose cm/s as common unit for all velocities (and tidal amplitudes) presented in the atlas. This is common practice in the field and allows for the most efficient and readable notation as almost all tidal band currents throughout the Arctic are of order O(1–10) cm/s.

A unique filename was created for each record consisting of region, mooring name, instrument type and deployment years (e.g. lapt_1893_ADCP300_2013-14).

### Tidal analysis

We analysed the current velocities using the T_TIDE Matlab toolbox^14^, which is based on tidal analysis methods described by Foreman^15^. T_TIDE performs a harmonic analysis based on the known frequencies for up to 69 tidal constituents and calculates all relevant tidal ellipse parameters (major and minor axis amplitudes, orientation, sense of rotation direction and phase) with their confidence intervals.

The number of resolvable constituents is determined by the length of the time series. In most ocean environments, the bulk of the total tidal variance is in eight constituents, four semidiurnal (M_2_, S_2_, K_2_, N_2_) and four diurnal (O_1_, K_1_, P_1_, Q_1_). Tidal analysis on shorter windows (<~180 days, as commonly available from temporary tide gauge deployments), report the combination of S_2_ and K_2_ as S_2_ only, while K_1_ and P_1_ are reported as K_1_. For barotropic tide heights, where amplitudes and phases are stable in time, these pairs can be separated in short records by “inference”^14,15^. In the present analysis, however, we expect that much of the tidal energy is in time-varying baroclinic modes where the assumptions required for inference may not apply.

The tidal parameters presented in this atlas are based on tidal analysis at each depth level over three different time periods: 30-day sliding windows (with original time increment), 90-day sliding windows (with 5-day increment) and the full record. Tidal parameters are reported for the midpoint of each window, rendering the temporal span of tidal parameters at each end 15 or 45 days shorter than the original time series for the 30- and 90-day analyses, respectively. T_TIDE is capable of dealing with some missing data at the cost of broadening confidence intervals. In practice, we chose to carry out tidal analysis only if less than 1/3 of the data within the window were missing to avoid large uncertainties.

The rationale for these three different approaches is as follows:

- 30-day sliding windows run over the whole record at each depth level. This method is used to identify short-term (monthly) variability of baroclinic tidal currents. A major caveat of this analysis is the potential effect of wind-driven inertial currents that may influence and even dominate tidal analysis in the upper ocean (see “Technical Validation”). Because of the unknown but potentially substantial influence of inertial currents on individual constituents, the user should not interpret upper ocean variability in this product as evidence of changing baroclinic tides (see technical validation below).
- 90-day analysis yields tidal parameters averaged over a longer period of time that reduces the influence of short-term synoptic wind influences as well as short-term changes of true baroclinic tidal currents on the harmonic analysis. This product is designed to allow for detailed analysis of individual tidal constituent ellipses and their variability over depth and time on seasonal to inter-annual time scales.
- Full-record analysis is carried out using all resolvable constituents and yields a single set of ellipse parameters for each. This provides robust, time-mean tidal information largely independent of short-term influences and thus represents barotropic and phase-locked baroclinic tides^16^. Note that the outcome of this analysis is not equivalent to averaging any of the previously discussed shorter-window analyses over the full record. Although differences in major axis amplitudes are often relatively small, other ellipse parameters (such as phase and orientation) may differ substantially. Additionally, full-record analysis is used to produce a “tidal prediction”; i.e., currents due to the combination of all T_TIDE-derived tidal constituents for the whole record at the original time increment (i.e. hourly in most cases).
- Supplementing harmonic tidal analysis, tidal band currents (TBC) are defined as currents that are band-pass filtered for periods between 10 h and 30 h. This comprises all semi-diurnal and diurnal tidal constituents as well as wind-driven inertial currents. This method does not require any averaging or smoothing over time and thus provides original amplitudes and variability for currents within the tidal band.

## Data Records

The atlas is archived as a collection of netCDF files, one for each instrumental record. Each file contains comprehensive metadata and a number of tidal variables as listed and shortly described in Table 1. The pathway to accessing the atlas is via the “table of inventory”, a human- and machine-readable table that provides relevant meta-information (file name, mooring name, region, start and end date, position, estimated bottom depth, instrument type, depth range covered and institution of origin), so that users can efficiently identify the records suitable for their needs. The data are accessible on the National Science Foundation Arctic Data Center (10.18739/A26M3340D^17^).

**Table 1:** List of variables in the Atlas (i.e. within each netCDF file).

Variable	Explanation
time	time vector at original increment
time_5day	time vector at 5-day increment
pressure	pressure vector with constant increment
pressure_flag	binary quality flag
six_con	list of the six tidal constituents used in T_TIDE analysis on 30-day and 90-day windows
all_con	list of all constituents used in full-record (FR) T_TIDE tidal analysis
latitude/longitude	location of the record
u_TBC/v_TBC	eastward/northward component of 10–30 h band-pass filtered Tidal Band Currents (TBC)
u_tide_pred/v_tide_pred	eastward/northward component of tidal prediction from FR analysis
maj_amp_30d/maj_amp_err_30d	major axis amplitude of six tidal constituents at all depth levels and at 5-day increment from 30-day analysis and the associated 95% confidence interval (‘err’)
min_amp_30d/min_amp_err_30d	minor axis amplitude of six tidal constituents at all depth levels and at 5-day increment from 30-day analysis and the associated 95% confidence interval (‘err’)
ori_30d/ori_err_30d	ellipse orientation of six tidal constituents at all depth levels and at 5-day increment from 30-day analysis and the associated 95% confidence interval (‘err’)
phase_30d/phase_err_30d	phase of six tidal constituents at all depth levels and at 5-day increment from 30-day analysis and the associated 95% confidence interval (‘err’)
maj_amp_90d/maj_amp_err_90d	as above, but for analysis on 90-day windows
min_amp_90d/min_amp_err_90d	as above, but for analysis on 90-day windows
ori_90d/ori_err_90d	as above, but for analysis on 90-day windows
phase_90d/phase_err_90d	as above, but for analysis on 90-day windows
maj_amp_FR/maj_amp_err_FR	as above, but for analysis on the full record (FR)
min_amp_FR/min_amp_err_FR	as above, but for analysis on the full record (FR)
ori_FR/ori_err_FR	as above, but for analysis on the full record (FR)
phase_FR/phase_err_FR	as above, but for analysis on the full record (FR)

## Technical Validation

### Instrument-related quality assessment

ADCP measurements close to the surface inherently suffer from contaminations due to surface reflections of sidelobe energy. This error is proportional to the cosine of the beam angle and the distance of the instrument to the surface and amounts to ~6% of the range for a typical 20° beam angle^18^. The effective range of contamination may be somewhat greater depending on the bin sizes. Since, for many records complete instrument information was unavailable, we chose to provide a mask blanking out the top 10% of the range of any records that reach the surface.

ADCP measurements depend on particles drifting in the water column that reflect the ADCP’s transmitted acoustic signal back to the instrument, where the Doppler shift of the signal is used to calculate velocities. However, in the relatively quiescent Arctic, the concentration of suspended particles can be very low, especially during winter when biological primary production effectively halts. With weak echoes, the ranges of ADCP profiles are substantially reduced: e.g. the nominal range for 300 kHz ADCPs exceeds 150 m but, in the Arctic, their effective range is ~50–60 m. Low backscatter amplitudes also lead to greater errors for speed and direction. Most records compiled in this atlas do not provide the extensive metadata to investigate this issue consistently, but erroneous data is commonly discarded in standard processing procedures.

### Influence of wind-driven inertial currents on tidal analysis

In this section, we motivate the use of different window-lengths within T_TIDE tidal analysis to partially mitigate the impact of wind-driven inertial currents on the analysis. Wind-driven inertial currents may substantially impact T_TIDE harmonic analysis in the Arctic^13^, where the local inertial period (12.735–11.967 h between 70° and 90° N) is very close to periods of the two major semidiurnal tidal constituents M_2_ (~12.421 h) and S_2_ (12.000 h). Baumann *et al*.^13^ demonstrated the impact of wind-driven inertial currents on tidal analysis using a damped-slab model^19,20^ with two different idealized mixed layer depths (10 m and 50 m). Effects are greatest for the 10-m SML case, which is representative of ice-free summers when surface mixed-layer depths are shallow. For the deeper 50-m case, the influence of wind-driven inertial currents is much reduced.

Here we use the same damped-slab model, for wind and ice conditions from model reanalysis products at a location offshore of the Laptev Sea continental slope, to demonstrate the different effects on 30-day, 90-day and full-time tidal analysis. After removing data recorded within 10% of the ocean surface from the ADCP depth, only 6% of the valid data are located within the top 10 m, whereas 54% lie between 10 and 50 m. We therefore consider the 50-m SML case of the slab model as the more representative condition for wind-forced inertial signals in the atlas.

**Fig. 2:**
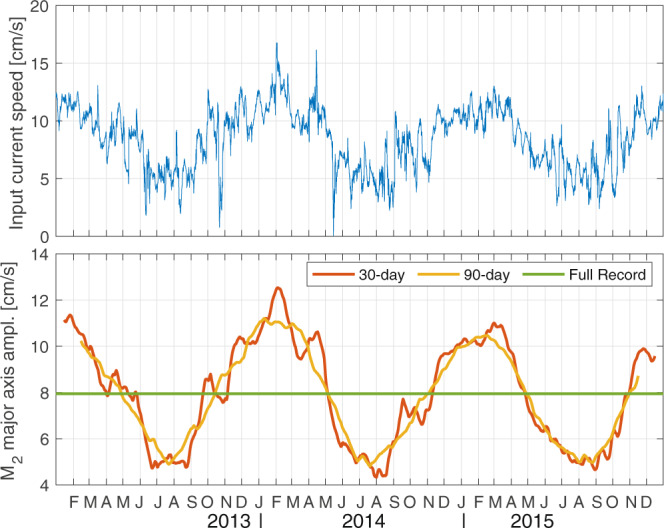
Tidal analysis using different window lengths performed on an artificial time series. The time series is constructed to resemble realistic conditions found at the eastern Eurasian continental slope (see Baumann *et al*.^13^) and consists of a complex harmonic oscillation at M_2_ frequency with an amplitude of 8 cm/s. The amplitude undergoes a seasonal cycle represented by a cosine function with 365.25-day period and amplitude of 3 cm/s. To this, we added inertial oscillations (average amplitude ~2 cm/s) simulated from a slab-model with 50-m SML (see^13^ for details). The 30-day and 90-day analyses predominantly follow the seasonal cycle, but noise has a substantial impact on the 30-day analysis. Some minor distortions of the seasonal signal are also visible for the 90-day analysis. The full-record analysis produces a single set of tidal ellipse parameters with the major axis amplitude almost exactly matching the input.

To demonstrate the influence of inertial noise on T_TIDE tidal analysis, we use an idealized tidal signal consisting of a complex harmonic oscillation at M_2_ frequency, whose amplitude (on average 8 cm/s) undergoes a seasonal variation (3 cm/s amplitude), similar to observed tides in the Laptev Sea and upper eastern Eurasian continental slope region (not shown). To this signal we added the simulated inertial currents from the slab model. We show the output of T_TIDE tidal analysis in Fig. 2. The 30-day analysis exhibits prominent high-frequency variability, which in this case is noise stemming from the inertial currents. As a consequence, the full range of M_2_ major axis amplitudes, a simple measure of variability over time, amounts to 8.2 cm/s which constitutes an overestimation of 37% compared to the expected seasonal variability of 6 cm/s. The 90-day analysis provides a clear seasonal cycle with major axis amplitudes exhibiting a range of 6.4 cm/s (i.e. 8% overestimation) and the full-records analysis provides amplitudes matching those of the input, despite variability through seasonality and wind-driven inertial currents. We note that, in conditions where inertial currents are continuously strong (more than half of tidal amplitude), inertial impacts are high on the 90-day and even full-record analysis as well. Although the dynamics are not well understood, we expect these conditions to be most significant close to the surface (within ~30 m) during ice-free summers.

### Influence of low frequency variability on tidal analysis

In the full record analysis, low frequency tidal constituents such as seasonal (SA) and semi-seasonal (SSA) are included depending of the length of the record. We cannot determine whether the energy ascribed to these constituents by T_TIDE harmonic fits is actually of tidal origin or merely follows seasonal variability caused by other physical drivers (analogous to the impact of inertial oscillations on semidiurnal constituents).

## Usage Notes

The wide range of possible applications (including climate modelling, fisheries, and offshore construction) requires high flexibility of the atlas. Using the “table of inventory” described in “Data Records”, users can easily identify the records useful to their specific task.

Large scale or pan-Arctic applications may benefit from a grouping of a number of (or all) records using clustering algorithms. Due to the strong dependence of tidal currents on topography, we recommend that clustering takes into account water depth as well as geographical location. An example of location and depth dependent clustering of all records is presented in Fig. 1. In the following section we use this clustering to illustrate pan-Arctic tidal current properties.

### Choosing the right atlas product

#### Full-record analysis. Comparison with barotropic tidal models

**Fig. 3:**
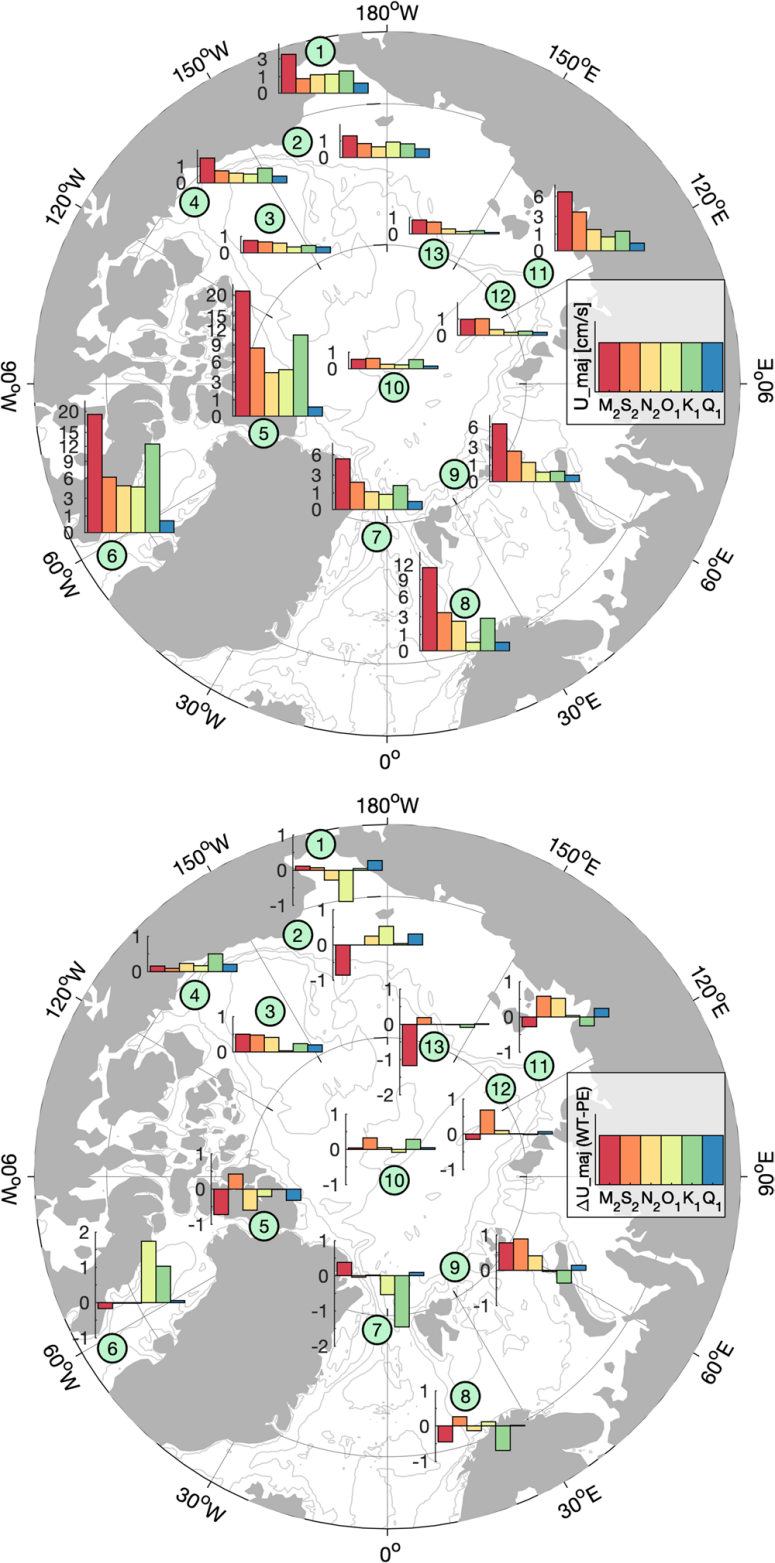
Major axis amplitudes of tidal constituents (U_maj_) from full-record analysis and their comparison to a barotropic tidal model. (top) Amplitudes are averaged vertically and over all records within each cluster. (bottom) Difference of U_maj_ for tidal constituents from full-record analysis and barotropic model output. Model data stems from Padman and Erofeeva^4^.

Barotropic tidal models are comparatively simple models that predict total tidal currents and current ellipse parameters for individual constituents from gravitational tidal forcing^4^. Despite their simplicity, results from these models are widely used in scientific research and many practical applications. The vertically averaged results of full-record tidal analyses from each ADCP record are the closest approximation to barotropic tidal currents in the atlas. Using the clustering shown in Fig. 1, the pan-Arctic spatial variability of major axis amplitudes from depth-averaged currents for the six most energetic diurnal and semidiurnal constituents shows that tidal current amplitudes and the relative contribution of the individual constituents vary widely across the Arctic (Fig. 3 top). Strongest tidal currents are observed in the Nares Strait (cluster *#5*) and Davis Strait (cluster *#6*), with M_2_ major axis amplitudes exceeding 20 cm/s followed by the diurnal K_1_ constituent with ~12 cm/s. Other regions of substantial tidal activity include the Barents Sea Opening (cluster #8) with ~12 cm/s for the leading M_2_ tide and very small diurnal contributions, the western Eurasian Basin continental slope (cluster #9) and the Laptev Sea (cluster #11) with dominating M_2_ amplitudes of 6–8 cm/s. While the Yermak Plateau is known for strong diurnal tidal currents^9^, in this visualization it is clustered together with Fram Strait moorings where the tidal signal is much weaker, yielding an average of only ~4 cm/s. Throughout deep basins (clusters #10, #12, #13 and #3) and the Pacific sector shelves and continental slopes (clusters #1, #2 and #4), tidal amplitudes for individual constituents are weaker (<4 cm/s) than those on the continental slopes on the Atlantic side.

These data may be used to validate barotropic tidal models. For example, differences between this atlas and output from the inverse barotropic tide model from Padman and Erofeeva^4^ (taken at the locations of every record in the atlas and performing the same averaging within each cluster) are relatively small (<2 cm/s, Fig. 3) with no systematic bias, and thus confirm the overall performance of that model.

#### 30-day and 90-day analyses: Spatio-temporal structure and variability of tidal parameters

**Fig. 4:**
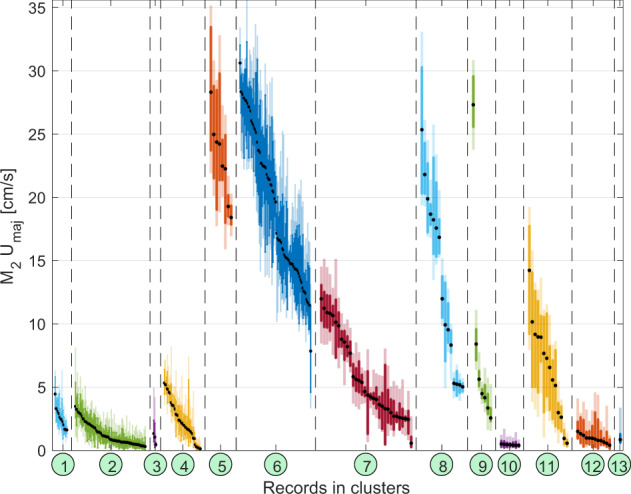
Spatio-temporal variability of tidal currents, illustrated by the range of M_2_ U_maj_ (from 30-day (light shading) and 90-day (solid colour) analysis, at 50 m depth) for each record in each cluster. The records within each cluster are sorted by average U_maj_ (black dots). For readability, horizontal plotting space was stretched for clusters with a smaller number of records (clusters #5 and #7-#13).

While barotropic tidal models provide general tidal information, which is invariant over depth and time, our new atlas provides information on the depth-dependence and temporal variability of tidal currents. The 30-day and 90-day analyses resolve the variability for individual tidal constituents on timescales from months to years, depending on the length of the record, including the seasonal cycle. Averaged over all records, the range of variability of M_2_ major axis amplitudes at 50 m depth amounts to 110% and 70% of the local mean (barotropic) major axis amplitude for the 30-day and 90-day analysis, respectively (or 4.8 cm/s and 2.8 cm/s in absolute terms) (Fig. 4). Even in regions with low average tidal amplitude (clusters #1–4), temporal variability of M_2_ tidal amplitudes can exceed 5 cm/s. Note that wind-driven inertial influence cannot be categorically excluded at 50 m depth. Smallest variability is found in cluster #10, centered at the North Pole, where tidal currents never exceed 2 cm/s (Fig. 4). Within the deep Beaufort Sea (cluster #3), records show a greater variability, sometimes exceeding 4 cm/s for the 30-day analysis. Generally, temporal variability of tidal currents is stronger in areas where tidal currents are strong, but the range of variations is smaller relative to the mean amplitude of tidal currents. For example, the average range of major axis amplitudes from 30-day analyses in cluster #5 is 9.3 cm/s, which is 40% of the mean amplitude (23 cm/s), whereas for cluster #10 an average range of 2.8 cm/s corresponds to 250% of the mean amplitude (1.1 cm/s, Fig. 4). A standout region for high variability of relatively strong tidal currents is cluster #11, comprising the Laptev Sea and the eastern Eurasian Basin continental slope and was extensively discussed in Baumann *et al*.^13^. Figure 4 further demonstrates that the difference between records within a cluster in most cases exceeds temporal variability within a record, highlighting the great spatial variability of tidal currents.

**Fig. 5:**
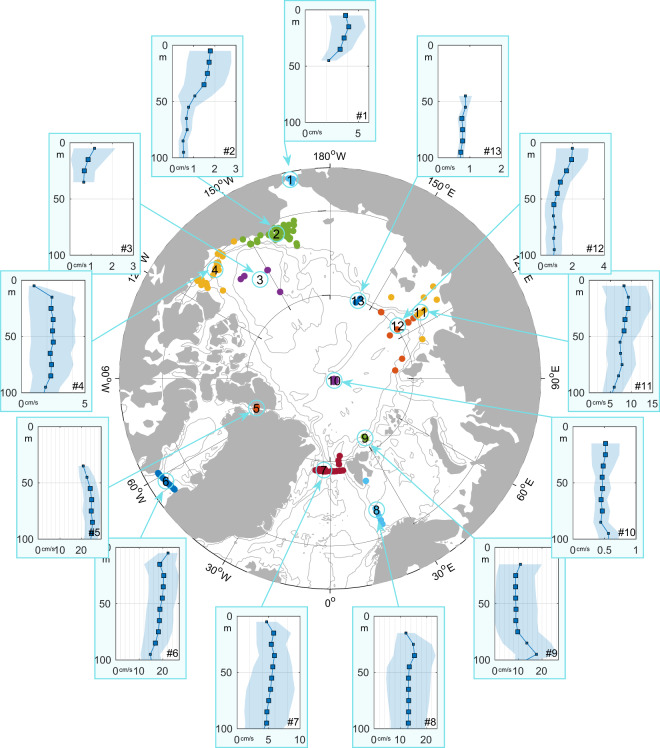
Cluster-average profiles of M_2_ major axis amplitudes from 90-day analysis over the top 100 m. Averages were taken over 10 m bins with squares in the profiles showing the center of the bins and the sizes reflect the relative number of measurements in that bin. Shading denotes ±1 standard deviation. The (linear) x-axis scales are different in each plot, but the vertical grid lines are always spaced by 2 cm/s.

The vertical structure (and thus shear) of baroclinic tidal currents is of major interest for the investigation of oceanic mixing processes. Tidal mixing can be important regionally^9,21^ and directly affects the sea ice cover in model simulations e.g.^3^. The vertical structure of M_2_ major axis amplitudes varies regionally across the Arctic (Fig. 5). While cluster-average profiles cannot be used to identify mixing processes, they may indicate the regional tendency for baroclinicity. While some regions exhibit vertical profiles with little vertical structure (clusters #4, #7, #8, #10 and #13), others show clear vertical gradients with surface amplification (clusters #1, #2, #3 and #12) or other structures (clusters #5, #6 and #11).

#### Tidal band currents and tidal prediction: High-frequency variability and kinetic energy

**Fig. 6:**
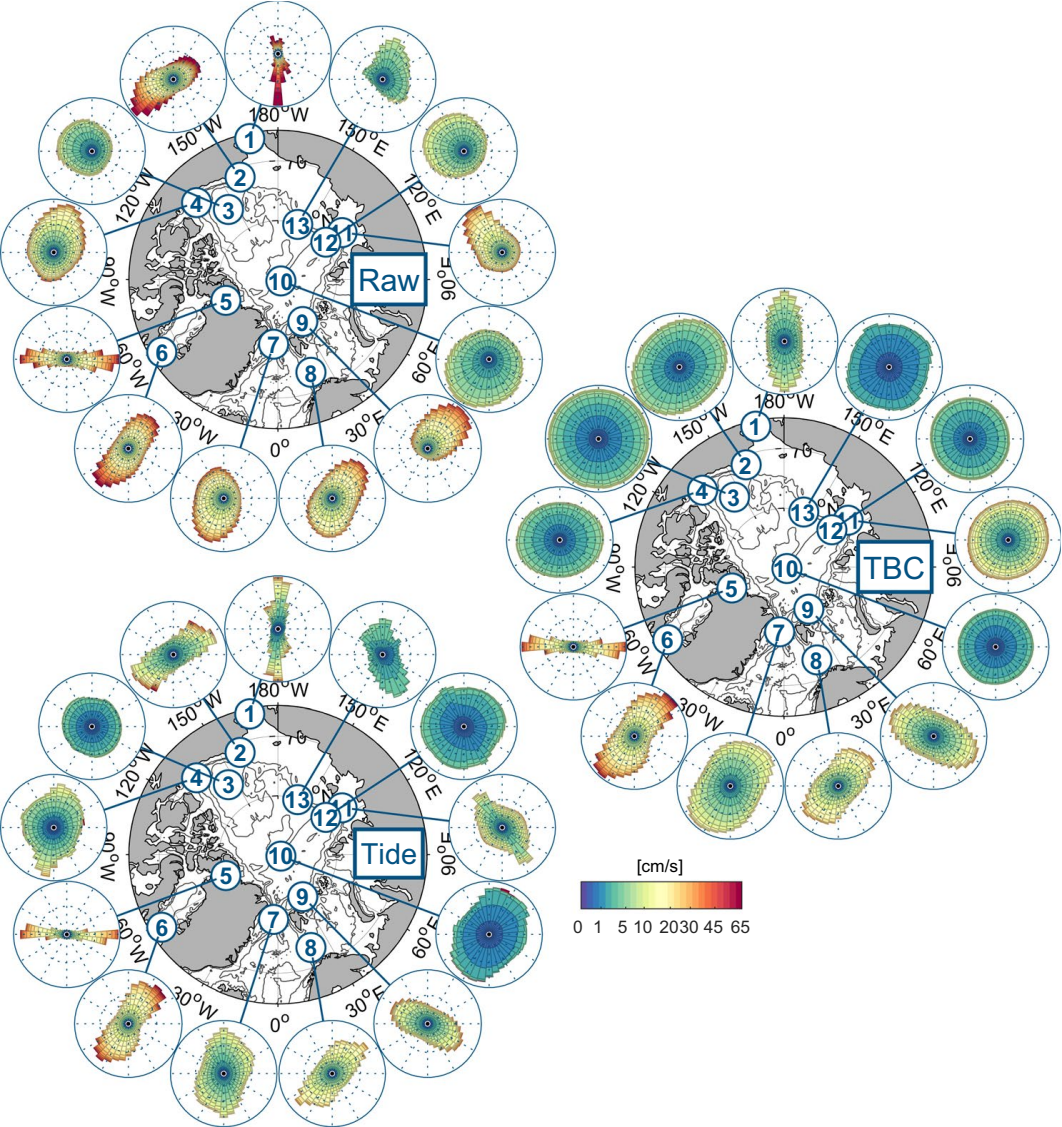
Regional current roses for observed raw currents (top), tidal band currents (TBC, right) and tidal prediction from full-record analysis (bottom). The roses are aligned with the true north of their respective centroid location (i.e. they fit in the map as they are without further rotation) and contain all observations within each cluster. The length of each 10° bin is proportional to the percentage of data within this bin. A nonlinear colour scale marks speed.

Tidal band currents (TBCs) provide the full spectrum of amplitudes and variability exerted by the combination of wind-driven inertial and tidal currents at diurnal and semidiurnal frequencies. Presently, there is no way to separate the wind-forced and tidal components analytically, so their properties have to be assessed jointly. The importance of TBCs relative to the full spectrum of observed raw currents across the Arctic can be seen in Fig. 6 (top and right). Despite regionally strong total currents in the Pacific sector of the Arctic (>50 cm/s at clusters #1, #2 and #3), TBCs are small throughout the region, barely reaching 10 cm/s. In the Atlantic sector, amplitudes of TBCs are often comparable to original, measured, (“raw”) current amplitudes (clusters #5, #6, #8 and #11), indicating that diurnal and semidiurnal tidal currents together with inertial currents are the defining characteristics of the dynamics in these regions. Figure 6 further reveals that TBCs can have a directional structure that fundamentally differs from the original raw currents (clusters #4, #7 and #9), likely caused by the interaction between tides and topographic features.

T_TIDE tidal current predictions based on full-record analysis include all tidal constituents that satisfy the Rayleigh criterion (i.e. that can be resolved given the length of the record)^15^. The prediction allows for the evaluation of amplitude and variability of current strength (or kinetic energy) ascribed to mean tidal currents by T_TIDE tidal analysis at any point in time of the record. Figure 6 shows that in regions where diurnal and semidiurnal tides are known to be strong, the tidal prediction is very similar to TBCs (compare clusters #5–9 in Fig. 6 bottom and right). In some regions, where tidal currents are known to be relatively weak, tidal prediction yields surprisingly strong currents (cluster #1, #2 and #4). This is due to the inclusion of low-frequency tidal constituents (e.g. seasonal (SA), semi-seasonal (SSA), monthly (Mm) and fortnightly (Mf)), which may coincide with other natural low-frequency drivers, such as the seasonal cycle of sea ice. Consequently, the absolute values should be treated with caution and the primary use may be the validation of equally processed output from numeric models.

#### Concluding remarks

Tidal currents play a vital role in the Arctic climate and ecosystems, but our understanding of their spatio-temporal variability is limited. The goal of our atlas of tidal currents described herein is to provide a tool that enables investigations into regional high-frequency dynamics in a changing Arctic Ocean. As a ground-truth for the modelling community, this may contribute to more reliable projections of future Arctic Ocean states. In order to maximize utility, we have provided different tidal products for different applications:

- Tidal harmonic parameters based on full-record analyses, e.g. for validation of barotropic tidal models.
- 90-day tidal analyses, e.g. for the analysis of seasonal to interannual variability of tidal currents.
- 30-day tidal analyses, e.g. for intra-annual variability. Users should be aware of the potentially dominating effect of wind-driven inertial currents on tidal parameters in this product.
- Tidal band currents (TBCs, band-pass filtered over 10–30 h), e.g. for analysis of high frequency variability without distinguishing between wind-driven inertial oscillations and tides.
- Tidal prediction for amplitude and variability of tidal currents as provided by T_TIDE tidal analysis.

## Online-only Table

**Online-only Table 1:** List of data sets used in the atlas.

Institute	Filenames in Atlas	DOI	Link/Citation
NPI	web_ATWAIN200_adcp150_2012-13	https://doi.org/10.21334/npolar.2017.73d0ea3a	https://doi.org/10.21334/npolar.2017.73d0ea3a ^22^
	web_ATWAIN800_adcp190_2012-13		
	web_ATWAIN800_adcp300_2012-13		
UAF/CFOS	chuk_Site1_adcp300_2008-09	N/A	https://data.nodc.noaa.gov/cgi-bin/iso?id=gov.noaa.nodc:0164964 ^23^
	chuk_Burger_adcp300_2008-09		
	chuk_Crackerjack_adcp300_2008-09		
	chuk_Site2_adcp300_2009-10		
	chuk_Site2_adcp300_2010-11		
	chuk_Burger_adcp300_2010-11		
	chuk_Crackerjack_adcp300_2010-11		
	chuk_CPAI01_adcp300_2011-12		
	chuk_CPAI02_adcp300_2011-12		
	chuk_Burger_adcp300_2011-12		
	chuk_Crackerjack_adcp300_2011-12		
	chuk_HS01_adcp300_2011-12		
	chuk_HS02_adcp300_2011-12		
	chuk_HS03_adcp300_2011-12		
	chuk_HS04_adcp300_2011-12		
	chuk_HS05_adcp300_2011-12		
	chuk_CPAI01_adcp300_2012-13		
	chuk_CPAI02_adcp300_2012-13		
	chuk_Burger_adcp300_2012-13		
	chuk_Crackerjack_adcp300_2012-13		
	chuk_Statoil3_adcp300_2012-13		
	chuk_Statoil4_adcp300_2012-13		
	chuk_Burger_adcp300_2013-14		
	chuk_Crackerjack_adcp300_2013-14		
NPI/WHOI	web_EA1_adcp75_2012-13	doi:10.18739/A2S569	https://arcticdata.io/catalog/view/doi:10.18739/A2S569 ^24^
	web_EA1_adcp300_2012-13		
	web_EA2_adcp75_2012-13		
	web_EA2_adcp300_2012-13		
	web_EA3_adcp75_2012-13		
	web_EA3_adcp300_2012-13		
	web_EA4_adcp75_2012-13		
	web_EA4_adcp300_2012-13		
IMR	bar_BSO3_a_rcm7_2006-07	N/A	Randi Ingvaldsen (randi.ingvaldsen@hi.no)
	bar_BSO3_d_rcm7_2006-07		
	bar_BSO4_a_rcm7_2006		
	bar_BSO4_d_rcm7_2006-07		
	bar_BSO5_a_rcm7_2006		
	bar_BSO5_d_rcm7_2006-07		
	bar_BSO2_a_rcm7_2007-08		
	bar_BSO2_d_rcm7_2007-08		
	bar_BSO3_a_rcm7_2007		
	bar_BSO3_d_rcm7_2007		
	bar_BSO4_a_rcm7_2007-08		
	bar_BSO4_d_rcm7_2007		
	bar_BSO5_a_rcm7_2007-08		
	bar_BSO5_d_rcm7_2007		
	bar_BSO2_d_rcm7_2007-08		
	bar_BSO2_a_rcm7_2007-08		
	bar_BSO3_d_rcm7_2007		
	bar_BSO3_a_rcm7_2007		
	bar_BSO4_d_rcm7_2007		
	bar_BSO4_a_rcm7_2007-08		
	bar_BSO5_d_rcm7_2007		
	bar_BSO5_a_rcm7_2007-08		
	bar_BSO2_d_rcm7_2008-09		
	bar_BSO2_a_rcm7_2008-09		
	bar_BSO3_d_rcm7_2008-09		
	bar_BSO3_a_rcm7_2008-09		
	bar_BSO4_d_rcm7_2008-09		
	bar_BSO4_a_rcm7_2008-09		
	bar_BSO5_d_rcm7_2008-09		
	bar_BSO5_a_rcm7_2008-09		
DFO	beau_CA10_adcp_2003-04	http://dx.doi.org/10.5884/11653	https://www.polardata.ca/pdcsearch/PDCSearch.jsp?doi_id=11792^25^
	beau_CA11_adcp_2003-04		
	beau_CA12_adcp_2003-04		
	beau_CA13_adcp_2003-05		
	beau_CA15_adcp_2003-04		
	beau_CA16_adcp_2003-04		
	beau_CA20_adcp_2003-04		
	beau_CA04_adcp_2003-04		
	beau_CA04_adcp_2002-03		
	beau_CA05_adcp_2003-04		
	beau_CA05_adcp_2002-03		
	beau_CA06_adcp_2002-03		
	beau_CA06_adcp_2003-04		
	beau_CA07_adcp_2003-04		
	beau_CA07_adcp_2002-03		
	beau_CA08_adcp_2003-04		
	beau_CA08_adcp_2002-03		
	beau_CA04-04_adcp_2004-05		
	beau_CA07-04_adcp_2004-05		
	beau_CA04_adcp300_2005-06		
	beau_CA05_adcp300_2005-06		
	beau_CA08_adcp300_2005-06		
	beau_CA05-08_adcp470_2008-09		
	beau_CA16-08_adcp470_2008-09		
CFOS/UAF	(beau_I2_backupfile)	N/A	N/A
CFOS	chuk_BC1_adcp_2010-11	N/A	https://data.nodc.noaa.gov/cgi-bin/iso?id=gov.noaa.nodc:0160090 ^26^
	chuk_BC2_adcp_2010-11		
	chuk_BC3_adcp_2010-11		
	chuk_BC4_adcp_2010-11		
	chuk_BC5_adcp_2010-11		
	chuk_BC6_adcp_2010-11		
	chuk_BC1_adcp_2011-12		
	chuk_BC2_adcp_2011-12		
	chuk_BC3_adcp_2011-12		
	chuk_BC4_adcp_2011-12		
	chuk_BC5_adcp_2011-12		
	chuk_BC2_adcp_2012-13		
	chuk_BC2_adcp_2013-14		
	chuk_BC2_adcp_2014-15		
	chuk_HSNE40_adcp_2012-13	N/A	https://data.nodc.noaa.gov/cgi-bin/iso?id=gov.noaa.nodc:0163833 ^23^
	chuk_HSNE50_adcp_2012-13		
	chuk_HSNE60_adcp_2012-13		
	chuk_HSNW40_adcp_2012-13		
	chuk_HSNW50_adcp_2012-13		
	chuk_HSNE40_adcp_2013-14		
	chuk_HSNE50_adcp_2013-14		
	chuk_HSNE60_adcp_2013-14		
	chuk_HSNW40_adcp_2013-14		
	chuk_HSNW50_adcp_2013-14		
	chuk_EBC_adcp_2010-11	doi:10.18739/A20K5X	https://search.dataone.org/view/doi:10.18739/A20K5X ^27^
	chuk_WBC_adcp_2010-11		
	chuk_EBC_adcp_2011-12	doi:10.18739/A21C7X	https://search.dataone.org/view/doi:10.18739/A21C7X ^28^
	chuk_WBC_adcp_2012-13		
	chuk_CEM_adcp_2014-15	N/A	N/A
APL	davis_C1_adcp_2004-05	doi:10.18739/A21S72	https://arcticdata.io/catalog/view/doi:10.18739/A21S72 ^29^
	davis_C2_adcp_2004-05		
	davis_C3_adcp_2004-05		
	davis_C4_adcp_2004-05		
	davis_C5_adcp_2004-05		
	davis_C6_adcp_2004-05		
	davis_WG1_adcp_2004-05		
	davis_C1_adcp_2005-06		
	davis_C2_adcp_2005-06		
	davis_C3_adcp_2005-06		
	davis_C5_adcp_2005-06		
	davis_WG3_adcp_2005-06		
	davis_WG1_adcp_2005-06		
	davis_BI4_adcp_2005-06		
	davis_C1_adcp_2006-07		
	davis_C2_adcp_2006-07		
	davis_C4_adcp_2006-07		
	davis_C6_adcp_2006-07		
	davis_WG3_adcp_2006-07		
	davis_WG1_adcp_2006-07		
	davis_BI3_adcp_2006-07		
	davis_C1_adcp_2007-08		
	davis_C2_adcp_2007-08		
	davis_C4_adcp_2007-08		
	davis_C5_adcp_2007-08		
	davis_C6_adcp_2007-08		
	davis_WG3_adcp_2007-08		
	davis_WG1_adcp_2007-08		
	davis_C3_adcp_2007-08		
	davis_C1_adcp_2008-09		
	davis_C2_adcp_2008-09		
	davis_C3_adcp_2008-09		
	davis_C4_adcp_2008-09		
	davis_C5_adcp_2008-09		
	davis_C6_adcp_2008-09		
	davis_WG4_adcp_2008-09		
	davis_WG1_adcp_2008-09		
	davis_BI4_adcp_2008-09		
	davis_C1_adcp_2009-10		
	davis_C2_adcp_2009-10		
	davis_C3_adcp_2009-10		
	davis_C4_adcp_2009-10		
	davis_C5_adcp_2009-10		
	davis_C6_adcp_2009-10		
	davis_WG1_adcp_2009-10		
	davis_WG4_adcp_2009-10		
	davis_BI4_adcp_2009-10		
	davis_C1_adcp_2010-11		
	davis_C2_adcp_2010-11		
	davis_C3_adcp_2010-11		
	davis_C4_adcp_2010-11		
	davis_C5_adcp_2010-11		
	davis_C6_adcp_2010-11		
	davis_WG1_adcp_2010-11		
	davis_WG4_adcp_2010-11		
	davis_BI4_adcp_2010-11		
	davis_WG15_adcp_2011-12		
	davis_WG1_adcp_2011-12		
	davis_WG4_adcp_2011-12		
	davis_BI4_adcp_2011-12		
	davis_C1_adcp_2011-13		
	davis_C2_adcp_2011-13		
	davis_C3_adcp_2011-13		
	davis_C4_adcp_2011-13		
	davis_C6_adcp_2011-13		
	davis_WG1_adcp_2012		
	davis_WG15_adcp_2012-13		
	davis_WG4_adcp_2012-13		
	davis_BI4_adcp_2012-13		
	davis_WG1_adcp_2013-14		
	davis_WG15_adcp_2013-14		
	davis_WG4_adcp_2013-14		
	davis_BI4_adcp_2013-14		
	davis_C1_adcp_2013-15		
	davis_C3_adcp_2013-15		
	davis_C5_adcp_2013-15		
	davis_C6_adcp_2013-15		
	davis_WG1_adcp_2014-15		
	davis_WG15_adcp_2014-15		
	davis_WG4_adcp_2014-15		
	davis_BI4_adcp_2014-15		
PMEL	chuk_ckp1a_adcp_2010-11	N/A	https://catalog.data.gov/dataset/noaa-ecofoci-chukchi-sea-adcp-mooring-time-series-data-stations-c1-c2-and-c3-2010-08-29-to-2012^30^
	chuk_ckp2a_adcp_2010-11		
	chuk_ckp3a_adcp_2010-11		
	chuk_ckp1a_adcp_2011-12		
	chuk_ckp2a_adcp_2011-12		
	chuk_ckp3a_adcp_2011-12		
HokU	chuk_B3_adcp300_2010-11	doi:10.18739/A2MT1D	https://arcticdata.io/catalog/view/doi:10.18739/A2MT1D^31^
	chuk_B1_adcp300_2010-11		
	chuk_B3_adcp300_2012-13		
	chuk_B1_adcp300_2013-14		
	chuk_B3_adcp300_2013-14		
	chuk_B1_adcp300_2009-10		
	chuk_B1_adcp300_2011-12		
	chuk_B3_adcp300_2009-10		
	chuk_B3_adcp300_2011-12		
NPI	fram_F12_adcp300_2013-14	https://doi.org/10.21334/npolar.2019.8bb85388	https://data.npolar.no/dataset/8bb85388-327e-4b01-892c-5d1836d44ca4^32^
	fram_F13_adcp300_2010-11		
	fram_F14_adcp300_2014-15		
	fram_F17_adcp300_2009-10		
	fram_F17_adcp300_2010-11		
	fram_F11_adcp300_2014-15		
	fram_F13_adcp300_2012-13		
	fram_F17_adcp300_2011-12		
	fram_F17_adcp300_2012-13		
	fram_F13_adcp300_2014-15		
	fram_F17_adcp300_2013-14		
	fram_F17_adcp300_2014-15		
	fram_F11_dcm_1998-99		
	fram_F12_dcm_1997-98		
	fram_F13_dcm_1997-98		
	fram_F14_dcm_2004-05		
	fram_F17_adcp300_2003-04		
	fram_F11_dcm_2000-02		
	fram_F12_dcm_1999-00		
	fram_F13_dcm_1998-99		
	fram_F14_dcm_2005-06		
	fram_F17_adcp300_2004-05		
	fram_F11_dcm_2002-03		
	fram_F12_dcm_2000-02		
	fram_F13_dcm_1999-00		
	fram_F14_rdcp600_2008-09		
	fram_F17_adcp300_2005-06		
	fram_F11_dcm_2003-04		
	fram_F12_dcm_2006-07		
	fram_F13_dcm_2003-04		
	fram_F17_adcp300_2006-07		
	fram_F11_rdcp600_2008-09		
	fram_F13_rdcp600_2008-09		
	fram_F17_adcp300_2008-09 fram_F14_rcm_1998-99		
	fram_F14_rcm_1999-00		
	fram_F14_rcm_2000-02		
	fram_F14_rcm_2002-03		
	fram_F14_rcm_2003-04		
	fram_F14_RDCP600_2009-10		
	fram_F14_RDCP600_2011-12		
CFOS/UAF	beau_I1_adcp300_2008-09	I1: doi:10.18739/A25S6X	https://arcticdata.io/catalog/view/doi:10.18739/A25S6X^33^
	beau_I1_adcp300_2008-09	I2: doi:10.18739/A2X911	https://arcticdata.io/catalog/view/doi:10.18739/A2X911^34^
	beau_I3_adcp300_2008-09	I3: doi:10.18739/A2233Z	https://arcticdata.io/catalog/view/doi:10.18739/A2233Z^35^
APL	ber_A2_adcp300_2014-15	N/A	http://psc.apl.washington.edu/HLD/Bstrait/Data/BeringStraitMooringDataArchive.html#Ascii_data^36,37^
	ber_A3_adcp300_2014-15		
	ber_A2W_adcp300_2007-08		
	ber_A2_adcp300_2007-08		
	ber_A4W_adcp300_2007-08		
	ber_A4_adcp300_2007-08		
	ber_A2W_adcp300_2008-09		
	ber_A2_adcp300_2008-09		
	ber_A4R_adcp300_2008-09		
	ber_A1_adcp300_2009-10		
	ber_A2W_adcp300_2009-10		
	ber_A2_adcp300_2009-10		
	ber_A3_adcp300_2009-10		
	ber_A4W_adcp300_2009-10		
	ber_A4_adcp300_2009-10		
	ber_A2W_adcp300_2010-11		
	ber_A3_adcp300_2010-11		
	ber_A4_adcp300_2010-11		
	ber_A4W_adcp300_2010-11		
	ber_A2W_adcp300_2011-12		
	ber_A4_adcp300_2011-12		
	ber_A2_adcp300_2015-16		
	ber_A3_adcp300_2015-16		
	ber_A4_adcp300_2015-16		
	ber_A2_adcp300_2013-14		
	ber_A3_adcp300_2013-14		
	ber_A4_adcp300_2014-15		
	ber_A3_adcp300_2007-08		
	ber_A1_adcp300_2008-09		
	ber_A3_adcp300_2008-09		
	ber_A2_adcp300_2010-11		
	ber_A2E_adcp300_2011-12		
	ber_A2_adcp300_2011-12		
	ber_A3_adcp300_2011-12		
WHOI	beau_D_adcp600_2005-06	N/A	https://www.whoi.edu/beaufortgyre
	beau_D_adcp600_2006-07		
	beau_D_adcp600_2007-08		
	beau_D_adcp600_2008-09		
	beau_A_adcp600_2010-11		
	beau_B_adcp600_2010-11		
	beau_D_adcp600_2010-11		
	beau_A_adcp600_2011-12		
	beau_B_adcp600_2011-12		
	beau_B_adcp600_2012-13		
	beau_D_adcp600_2012-13		
	beau_A_adcp600_2013-14		
	beau_B_adcp600_2013-14		
	beau_D_adcp600_2013-14		
	beau_A_adcp600_2014-15		
	beau_B_adcp600_2014-15		
	beau_D_adcp600_2014-15		
	beau_A_adcp600_2015-16		
	beau_B_adcp600_2015-16		
	beau_D_adcp600_2015-16		
UDel	cp_KS02_adcp75_2003-06	doi:10.18739/A23K67	https://arcticdata.io/catalog/view/doi:10.18739/A23K67^38^
	cp_KS10_adcp75_2003-06		
	cp_KS12_adcp75_2003-06		
	cp_KS14_adcp75_2003-06		
	cp_KS04_adcp75_2007-09	doi:10.18739/A2RR77	https://arcticdata.io/catalog/view/doi:10.18739/A2RR77^39^
	cp_KS06_adcp75_2007-09		
	cp_KS08_adcp75_2007-09		
	cp_KS10_adcp75_2007-09		
	cp_KS12_adcp75_2007-09		
CFOS/UAF		https://doi.org/10.5065/D62805QS	https://data.eol.ucar.edu/dataset/62.316^40^
	chuk_CC1_adcp_2003-04		
	chuk_Camden_adcp_2004-07	N/A	N/A^41,42^
	chuk_Dinkum_adcp_2004-06		
	chuk_Smith_adcp_2004-05		
IARC/UAF	eeb_M3e_adcp75_2013-15	doi:10.18739/A2RS9B	https://arcticdata.io/catalog/view/doi:10.18739/A2RS9B^43^
	eeb_M6b_adcp75_2013-15		
	eeb_M1_1a_adcp75_2013-15 eeb_M1_2a_adcp300_2013-15		
	eeb_M1_3a_adcp300_2013-15		
	eeb_M1_4a_adcp300_2013-15		
	eeb_M1_5a_adcp300_2013-14		
	eeb_M1_6a_adcp300_2013-15		
	eeb_M3e_adcp300_2013-15		
	eeb_M6b_adcp300_2013-15		
	eeb_M1_1b_adcp75_2015-18	doi:10.18739/A2HT2GB80	https://arcticdata.io/catalog/view/doi:10.18739/A2HT2GB80^44^
	eeb_M1_2b_adcp300_2015-18		
	eeb_M1_2b_adcp75_2015-18		
	eeb_M1_3b_adcp300_2015-18		
	eeb_M1_4b_adcp300_2015-18		
	eeb_M1_4b_adcp75_2015-18		
	eeb_M1_5b_adcp300_2015-18		
	eeb_M3f_adcp300_2015-18		
	eeb_M3f_adcp75_2015-18		
	eeb_M1c_adcp300_2004-05	N/A	https://nabos.iarc.uaf.edu/NABOS/data/registered/main.php
	eeb_M3a_adcp_2004-05		
	eeb_M1d_adcp_2005-06		
	eeb_M3b_adcp_2005-06		
	eeb_M1e_adcp_2006-07		
	eeb_M3c_adcp_2006-07		
	eeb_M5b_adcp_2006-07		
	mb_M9a_adcp_2007-08		
	mb_M10a_adcp_2007-08		
	eeb_M1g_adcp300a_2008-10		
	eeb_M1g_adcp300b_2008-10		
	eeb_M1g_adcp300c_2008-10		
	web_M4c_adcp300_2009-10		
UiB	web_Y1_comb_2014-15	https://doi.org/10.21335/NMDC-1508183213	http://metadata.nmdc.no/metadata-api/landingpage/ac35993ab477cb6b7d4a0d0070d57b43^45^
	web_Y2_comb_2014-15		
AWI	fram_F3-15_adcp_2012-15	https://doi.pangaea.de/10.1594/PANGAEA.853902	https://doi.pangaea.de/10.1594/PANGAEA.853902^46^
	fram_F4-15_adcp_2012-15	https://doi.pangaea.de/10.1594/PANGAEA.853903	https://doi.pangaea.de/10.1594/PANGAEA.853903^47^
	fram_F5-15_adcp_2012-15	https://doi.pangaea.de/10.1594/PANGAEA.853904	https://doi.pangaea.de/10.1594/PANGAEA.853904^48^
	fram_F7-12_adcp_2012-15	https://doi.pangaea.de/10.1594/PANGAEA.853905	https://doi.pangaea.de/10.1594/PANGAEA.853905^49^
	fram_F8-13_adcp_2012-14	https://doi.pangaea.de/10.1594/PANGAEA.853907	https://doi.pangaea.de/10.1594/PANGAEA.853907^50^
	fram_F10-12_adcp_2012-15	https://doi.pangaea.de/10.1594/PANGAEA.853910	https://doi.pangaea.de/10.1594/PANGAEA.853910^51^
	fram_F15-9_adcp_2012-14	https://doi.pangaea.de/10.1594/PANGAEA.853918	https://doi.pangaea.de/10.1594/PANGAEA.853918^52^
	fram_F16-9_adcp_2012-14	https://doi.pangaea.de/10.1594/PANGAEA.853920	https://doi.pangaea.de/10.1594/PANGAEA.853920^53^
AWI	lapt_1893_adcp600_2013-14	https://doi.pangaea.de/10.1594/PANGAEA.908837	https://doi.pangaea.de/10.1594/PANGAEA.908837^54^
	lapt_1893_adcp1200_2014-15		
	lapt_1893_adcp300_2013-14		
	lapt_1893_adcp300_2014-15		
	lapt_Lena03_adcp300_2003-04		
	lapt_Lena98_adcp300_1998-99		
	lapt_OSL2a_adcp300_2005-06		
	lapt_OSL2a_adcp300_2006-07		
	lapt_OSL2e_adcp300_2010-11		
	lapt_OSL2f_adcp300_2011-12		
	lapt_OSL3_adcp300_2008-09		
	lapt_Yana98_adcp300_1998-99		
	eeb_Vilk_adcp300_2013-14		
	eeb_Vilk_adcp150_2013-14		
APL	pole_npeo_adcp300_2001-02	doi:10.5065/D6P84921	https://arcticdata.io/catalog/view/doi:10.5065/D6P84921^55^
	pole_npeo_adcp300_2002-03		
	pole_npeo_adcp300_2003-04		
	pole_npeo_adcp300_2004-05		
	pole_npeo_adcp300_2005-06		
	pole_npeo_adcp300_2006-08		
	pole_npeo_adcp300_2008-10		
WHOI	beau_bs2_adcp_2002-03	doi:10.5065/D6J964FR	https://arcticdata.io/catalog/view/doi:10.5065/D6J964FR^56^
	beau_bs3_adcp_2002-03		
	beau_bs4_adcp_2002-03		
	beau_bs5_adcp_2002-03		
	beau_bs6_adcp_2002-03		
	beau_bs1_adcp_2003-04	doi:10.5065/D6FF3QF3	https://arcticdata.io/catalog/view/doi:10.5065/D6FF3QF3^57^
	beau_bs2_adcp_2003-04		
	beau_bs3_adcp_2003-04		
	beau_bs4_adcp_2003-04		
	beau_bs5_adcp_2003-04		
	beau_bs6_adcp_2003-04		
IMR	bar_BSO1_adcp150_2005-06	N/A	Randi Ingvaldsen (randi.ingvaldsen@hi.no)
	bar_BSO1_adcp150_2006-07		
	bar_BSO1_adcp75_2007-08		
APL	chuk_SBI3_adcp300_2002-03	doi:10.5065/D64747X3	https://arcticdata.io/catalog/view/doi:10.5065/D64747X3^58^
	chuk_SBI3_adcp300_2003-04		
	chuk_SBI4_adcp300_2002-03		
	chuk_SBI4_adcp300_2003-04		
UiB	bar_Stor_adcp300_2003-04	https://doi.org/10.21335/NMDC-273201156	http://metadata.nmdc.no/metadata-api/landingpage/43cf0f5690de61c093ee18010c65f8e7^59^
	bar_Stor_adcp300_2004-05		
	bar_Stor_adcp300_2005-06		
	bar_Stor_adcp300_2006-07		
IARC/UAF	bar_StA_adcp300a_2009-10	N/A	N/A
	bar_StA_adcp300b_2009-10		
UPMC/LOCEAN	web_YP_adcp75_2007-08	doi:10.17882/51023	https://www.seanoe.org/data/00399/51023/^60^
INRS-ETE	beau_CA04-04_adcp_2004-05	N/A	www.polardata.ca/pdcsearch/PDCSearchDOI.jsp?doi_id=11792^25^
	beau_CA07-04_adcp_2004-05		
	beau_CA04_adcp300_2005-06		
	beau_CA05_adcp300_2005-06		
	beau_CA08_adcp300_2005-06		
	beau_CA05-08_adcp470_2008-09		
	beau_CA16-08_adcp470_2008-09		
ULaval	beau_BRK-15_adcp_2015-16	https://doi.org/10.5884/13107	www.polardata.ca/pdcsearch/PDCSearchDOI.jsp?doi_id=13107^61^
	beau_BRG-15_adcp75_2015-16		
	beau_BRG-15_adcp150_2015-16		
	beau_BR3-15_adcp75_2015-16		
	beau_BR1-15_adcp150_2015-16		
	beau_BR1-15_adcp75_2015-16		
	beau_BRK-16_adcp_2016-17		
	beau_BR1-16_adcp150_2016-17		
	beau_BRG-16_adcp75_2016-17		
	beau_BR1-16_adcp75_2016-17		
	beau_BRG-16_adcp150_2016-17		
ULaval	beau_BR-2_adcp300_2011-12	N/A	www.polardata.ca/pdcsearch/PDCSearchDOI.jsp?doi_id=11654^62^
	beau_BR-A_adcp75_2011-12		
	beau_BR-A_adcp150_2011-12		
	beau_BR-B_adcp150_2011-12		
	beau_BR-G_adcp75_2011-12		
	beau_BR-01_adcp75_2012-13		
	beau_BR-02_adcp300_2012-13		
	beau_BR-B_adcp300_2012-13		
	beau_BR-G_adcp75_2012-13		
